# Low-intensity daily smoking and cause-specific mortality in Mexico: prospective study of 150 000 adults

**DOI:** 10.1093/ije/dyab013

**Published:** 2021-02-28

**Authors:** Blake Thomson, Roberto Tapia-Conyer, Ben Lacey, Sarah Lewington, Raúl Ramirez-Reyes, Diego Aguilar-Ramirez, Louisa Gnatiuc, William G Herrington, Jason Torres, Eirini Trichia, Rachel Wade, Rory Collins, Richard Peto, Pablo Kuri-Morales, Jesus Alegre-Díaz, Jonathan R Emberson

**Affiliations:** 1 Clinical Trial Service Unit and Epidemiological Studies Unit (CTSU), Nuffield Department of Population Health (NDPH), University of Oxford, Oxford, UK; 2 School of Medicine, National Autonomous University of Mexico (UNAM), Mexico City, Mexico; 3 MRC Population Heath Research Unit, CTSU, NDPH, University of Oxford, Oxford, UK; 4 UKM Medical Molecular Biology Institute (UMBI), Universiti Kebangsaan Malaysia, Kuala Lumpur, Malaysia

**Keywords:** Smoking, cause-specific mortality, cohort study, Mexico

## Abstract

**Background:**

Research is needed to determine the relevance of low-intensity daily smoking to mortality in countries such as Mexico, where such smoking habits are common.

**Methods:**

Prospective study of 159 755 Mexican adults recruited from 1998–2004 and followed for cause-specific mortality to 1 January 2018. Participants were categorized according to baseline self-reported smoking status. Confounder-adjusted mortality rate ratios (RRs) at ages 35–89 were estimated using Cox regression, after excluding those with previous chronic disease (to avoid reverse causality).

**Results:**

Among 42 416 men and 86 735 women aged 35–89 and without previous disease, 18 985 men (45%) and 18 072 women (21%) reported current smoking and 8866 men (21%) and 53 912 women (62%) reported never smoking. Smoking less than daily was common: 33% of male current smokers and 39% of female current smokers. During follow-up, the all-cause mortality RRs associated with the baseline smoking categories of <10 cigarettes per day (average during follow-up 4 per day) or ≥10 cigarettes per day (average during follow-up 10 per day), compared with never smoking, were 1.17 (95% confidence interval 1.10–1.25) and 1.54 (1.42–1.67), respectively. RRs were similar irrespective of age or sex. The diseases most strongly associated with daily smoking were respiratory cancers, chronic obstructive pulmonary disease and gastrointestinal and vascular diseases. Ex-daily smokers had substantially lower mortality rates than those who were current daily smokers at recruitment.

**Conclusions:**

In this Mexican population, low-intensity daily smoking was associated with increased mortality. Of those smoking 10 cigarettes per day on average, about one-third were killed by their habit. Quitting substantially reduced these risks.

Key Messages

- Low-intensity daily smoking is common in many countries but little evidence exists on its relevance to mortality.
- In this large study of Mexican adults, half of men and one-quarter of women smoked, but average cigarette consumption was low and non-daily smoking common.
- Smoking even a few cigarettes per day was associated with increased mortality.
- As in other populations, quitting smoking avoided much of the excess risk of death associated with prolonged smoking.

## Introduction

Smoking is a leading preventable cause of death,^1^ with studies in high-income countries showing that it kills up to two-thirds of those who smoke.^2–5^ According to the Global Burden of Disease Study, smoking caused nearly 9 million deaths in 2019.^6^ However, there is limited large-scale prospective evidence on smoking from low- or middle-income countries (LMICs), where the majority of the world’s smokers live^1^ and where smoking habits may differ from those in high-income countries. In particular, in many LMICs, low-intensity smoking (i.e. smoking relatively few cigarettes per day) is common,^7^ but the health effects of low-intensity smoking are not well established.

Of the approximately 50 million smokers in the Latin American and Caribbean region,^8^ approximately 10 million live in Mexico. National surveys from Mexico indicate that non-daily smoking is common (fewer than half smoke daily) and even among those who do smoke daily, the average number of cigarettes smoked per day is less than 10.^9^^,^^10^ This is in contrast with smoking habits in most previously studied high-income populations, where daily smokers may typically smoke between 15 and 20 cigarettes per day.^4^^,^^5^ Data from the US National Health Interview Survey 2018^11^ also reveal markedly different smoking patterns between Mexican-Americans and other smokers, with about two-fifths of Mexican-American smokers reporting smoking less than daily compared with only one-fifth of non-Hispanic White US smokers. In addition, consumption among daily smokers in that study was eight cigarettes per day for Mexican-American smokers compared with 15 cigarettes per day for non-Hispanic White smokers. However, to date, assessments of the impact of such low-intensity smoking habits on mortality among Mexicans or Mexican-Americans have been limited to indirect methods.^1^^,^^12^

The aim of this paper is to estimate the effect of low-intensity daily smoking on cause-specific mortality using data from the Mexico City Prospective Study of 150 000 adults who were recruited from 1998 to 2004 and followed until 2018.^13^

## Methods

Approval for the study was given by the Mexican Ministry of Health, the Mexican National Council of Science and Technology (0595 P-M) and the Central Oxford Research Ethics Committee (C99.260). All study participants provided written informed consent.

### Recruitment

From 1998–2004, 52 644 men and 107 111 women aged 35 years or older, from two districts of Mexico City, were visited in their homes and agreed to enrol in a prospective study (at least one participant was recruited from 94% of eligible households).^13^ Trained nurses recorded age, sex, socioeconomic status, lifestyle factors (including smoking status, amount smoked, age began smoking and information on quitting), current medications and medical history. Blood pressure, weight, height, waist circumference and hip circumference were measured and a 10-mL blood sample was collected.

### Resurvey assessment

From 2015–19, a resurvey was performed in which the households within randomly-selected streets within the two study districts were revisited. A total of 10 144 surviving participants were revisited and agreed to take part in the resurvey (which involved repeat questionnaire data, physical measurements and the collection of blood and urine samples). The main purpose of the resurvey was to estimate the extent to which risk exposures (such as smoking habits) varied over time, in order to ensure that prospective analyses derived from baseline-defined categories could be properly analysed and interpreted.

### Mortality follow-up

Mortality was tracked to 1 January 2018 through probabilistic linkage (based on participant name, age and sex) to the national death register, which encodes all diseases listed on the death certificate according to the International Classification of Diseases, 10th Revision (ICD-10). Study clinicians then reviewed and, where necessary, recoded the underlying cause of death.^14^ Details of the ICD-10 codes contributing to each analysed cause of death are provided in Table S1, available as Supplementary data at *IJE* online.

### Statistical analysis

To minimize reverse causality bias (i.e. where previous diseases lead to a change, such as a reduction in smoking habits preceding recruitment), analyses excluded individuals with known cardiovascular disease, cirrhosis, cancer, emphysema, chronic kidney disease or diabetes. (Analyses including those with prior disease are included in the Supplementary Figure S6, available as Supplementary data at *IJE* online.) Participants who had missing or implausible covariate data (see below), uncertain mortality linkage or who were aged 90 years or older were also excluded. Remaining participants were classified into five baseline-defined smoking groups: never smoker, ex-smoker, current non-daily smoker, current daily smoker of <10 cigarettes per day, and current daily smoker of ≥10 cigarettes per day. In some analyses, daily smokers were subdivided by age started (<18 years versus ≥18 years) instead of amount smoked, and ex-smokers subdivided into ex-daily smokers versus ex- non-daily smokers.

Cox regression was used to estimate adjusted mortality rate ratios (RRs) associated with each baseline-defined smoking category. Most analyses were for deaths at ages 35 to 89 years, with participants surviving to age 90 censored on the date of their 90th birthday. The RRs were adjusted for age at risk (in 5-year age groups), location (two districts), highest education attained (university or college, high school, elementary school or other), alcohol consumption (never, former, less than monthly, up to 2 days per week or more than 2 days per week) and sex (where appropriate). For each category of current daily smoking at recruitment, we plotted RRs by amount smoked against the mean number of cigarettes smoked per day at resurvey (calculated among participants who continued to smoke). This was done to relate the RRs in each of the baseline-defined smoking categories to an estimate of the usual (i.e. long-term average) number of cigarettes smoked per day during follow-up in these categories. In the figures, every RR (including the reference group of never smokers assigned an RR of 1.0) is shown with a group-specific confidence interval (CI) that reflects the amount of information only in that single category.^15^ In the text, conventional 95% CIs comparing two categories directly are used (e.g. daily vs never smoking). The RR for daily vs never smokers was used to estimate the proportion of deaths among daily smokers attributable to smoking, through the equation (RR—1)/RR.

Findings from the present Mexican cohort subsequently led to re-examination in US data^11^ of the association between smoking and mortality in different races, comparing associations among Mexican-Americans, other Hispanics and non-Hispanic groups (further details on the background, methods and results are in the Supplementary material).

Analyses were conducted using SAS version 9.4 (SAS Institute) and R version 3.1.1 (www.r-project.org/).

## Results

### Participants

Among the 112 333 eligible households visited, 106 059 agreed to participate, yielding a total of 159 755 participants. Of these, 854 (0.5%) were excluded because they were aged ≥90 years at recruitment, a further 27 940 (17.5%) were excluded because they had previous disease (mainly diabetes) and a further 1810 (1.1%) were excluded because of missing exposure, covariate or outcome data. The remaining 129 151 participants included 42 416 men (mean age 52 years) and 86 735 women (mean age 50 years) (Table 1).

**Table 1:** Characteristics of men and women aged 35–89 years and without previous chronic disease at recruitment, by baseline-defined smoking status

	Men (*n *=* *42 416)					Women (*n *=* *86 735)				
			Current smoker (*n *=* *18 985)					Current smoker (*n *=* *18 072)		
	Never smoker (*n *=* *8866)	Ex-smoker (*n *=* *14 565)	Less than daily (*n *=* *6177)	Daily <10/day (*n *=* *8064)	Daily ≥10/day (*n *=* *4744)	Never smoker (*n *=* *53 912)	Ex- smoker (*n *=* *14 751)	Less than daily (*n *=* *7043)	Daily <10/day (*n *=* *8498)	Daily ≥10/day (*n *=* *2531)
Smoking behaviour										
Mean cigarettes/day^a^	–	–	0.2 (0.5)	4.1 (2.2)	16.3 (7.5)	–	–	0.1 (0.3)	3.7 (2.0)	14.5 (6.0)
Age started smoking (years)	–	17.6 (5.7)	18.9 (6.4)	18.2 (6.6)	16.8 (5.3)	–	22.0 (8.3)	23.8 (9.5)	21.6 (8.5)	19.8 (7.0)
Ever tried to quit	–	–	40%	43%	34%	–	–	42%	53%	43%
Inhale smoke	–	–	64%	76%	84%	–	–	52%	68%	76%
Others smoke inside home	16%	19%	26%	34%	42%	30%	33%	44%	55%	60%
Age (years)	52 (13)	55 (14)	47 (10)	50 (12)	50 (11)	52 (13)	50 (12)	45 (9)	46 (10)	48 (10)
Resident in Iztapalapa	57%	56%	59%	56%	53%	64%	57%	60%	56%	48%
Education attained										
University	32%	24%	26%	21%	24%	10%	17%	16%	17%	22%
High school	24%	24%	33%	30%	29%	22%	28%	36%	34%	33%
Elementary school	35%	42%	37%	41%	39%	51%	44%	42%	43%	39%
Other	9%	10%	5%	8%	7%	16%	11%	6%	6%	6%
Drinking behaviour										
Drink at least weekly	48%	46%	51%	48%	46%	51%	57%	66%	61%	56%
Drink less than weekly	29%	41%	35%	39%	41%	13%	22%	16%	19%	22%
Former drinker	9%	8%	11%	10%	9%	3%	5%	8%	8%	8%
Never drinker	14%	5%	2%	3%	4%	33%	16%	10%	12%	14%
Anthropometry and blood pressure										
BMI (kg/m²)	28.0 (4.3)	28.3 (4.2)	28.6 (4.3)	27.6 (4.4)	26.9 (4.4)	29.6 (5.2)	29.8 (5.4)	29.7 (5.3)	28.9 (5.2)	27.9 (5.3)
Waist-to-hip ratio	0.95 (0.07)	0.96 (0.07)	0.95 (0.06)	0.95 (0.06)	0.95 (0.07)	0.88 (0.07)	0.87 (0.07)	0.87 (0.07)	0.86 (0.07)	0.86 (0.07)
Systolic BP (mmHg)	128 (16)	129 (16)	127 (14)	127 (15)	127 (15)	127 (17)	125 (16)	122 (15)	122 (15)	123 (15)
Diastolic BP (mmHg)	84 (10)	85 (10)	84 (10)	84 (10)	84 (10)	83 (10)	82 (10)	81 (10)	80 (10)	81 (10)

Mean (standard deviation) or percent shown. Participants with previously diagnosed chronic disease (chronic kidney disease, ischaemic heart disease, stroke, cirrhosis, cancer, emphysema or diabetes) are excluded.

BMI, body mass index; BP, blood pressure.

a Cigarettes per day among non-daily smokers estimated by calculating the number of cigarettes smoked per occasion multiplied by smoking frequency (e.g. daily, weekly) to determine average cigarettes smoked per day over a 1-month period.

### Smoking and other baseline characteristics

**Figure 1:**
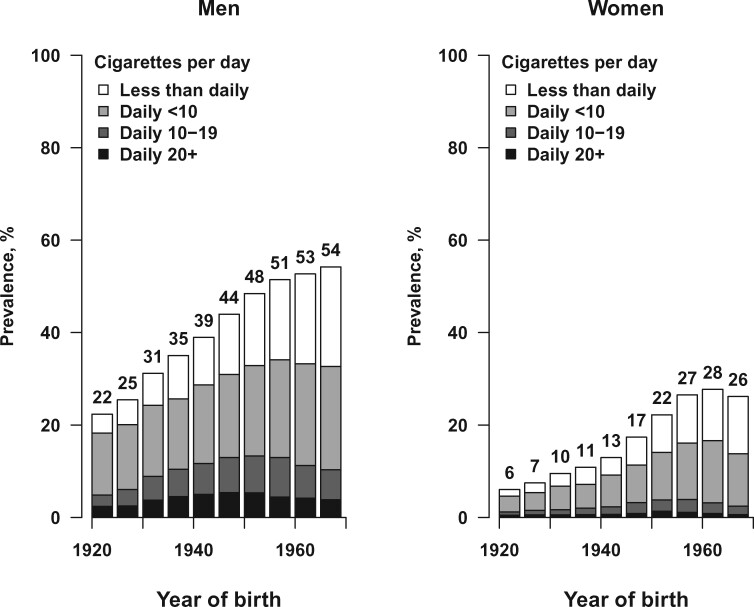
Current smoking by year of birth. Ten 5-year birth cohorts are shown, from 1920-24 to 1965-69

Of the 42 416 men, 18 985 (45%) reported being a current smoker, 14 565 (34%) reported being an ex-smoker and 8866 (21%) reported being a never smoker at recruitment. Of the male current smokers, 12 808 (67%) reported smoking daily (mean cigarettes smoked per day at recruitment: 8.6, mean age started: 18 years) and 6177 (33%) reported smoking less than daily. Of the 86 735 women, 18 072 (21%) reported being a current smoker, 14 751 (17%) reported being an ex-smoker and 53 912 (62%) reported being a never smoker. Of the female current smokers, 11 029 (61%) reported smoking daily (mean cigarettes smoked per day at recruitment: 6.1, mean age started: 21 years) and 7043 (39%) reported smoking less than daily. Figure 1 shows the smoking patterns among male and female current smokers at recruitment, by birth cohort.

The prevalence of ever smoking among men was similar across all birth cohorts, but among women ever smoking was more common in the more recent birth cohorts (Supplementary Figure S1, available as Supplementary data at *IJE* online). Smoking cessation was positively associated with age in both men and women and, at recruitment, nearly half of both male and female ever-smokers had quit. Among current smokers, the mean number of cigarettes smoked per day was generally similar across birth cohorts for both sexes (Supplementary Figure S2, available as Supplementary data at *IJE* online). The mean age started was approximately 18 years for all male birth cohorts, but for female birth cohorts the mean age started smoking decreased substantially from 27 years among women born before 1930 to 19 years among women born after 1965 (Supplementary Figure S2).

Men who smoked daily were less likely to have completed university than men who had never smoked; the reverse was true for women (Table 1). Compared with daily smokers, men and women who smoked less than daily were younger, less likely to inhale while smoking and less likely to live with a smoker. In both men and women, daily smokers had slightly lower mean body mass index (BMI) than never smokers. On average, those who smoked 10 or more cigarettes per day started at a younger age, had a lower BMI and were less likely to have tried to quit smoking than those who smoked fewer than 10 cigarettes per day. Among the 29 316 ex-smokers, 11 462 (39%) had previously smoked less than daily and 4940 (17%) had quit within the 3 years before recruitment (Supplementary Table S2).

### Smoking habits at resurvey

Of the 129 151 participants, 8753 were among the 10 144 who were resurveyed in 2015–19 (Supplementary Table S3, available as Supplementary data at *IJE* online). Of these 8753, 1432 had been daily smokers at baseline (1998–2004), but by resurvey (2015–19) just 672 (47%) reported still smoking daily, 141 (10%) reported smoking less than daily and 537 (38%) reported having quit (often because of illness). Among those who smoked <10 cigarettes per day at baseline and continued to smoke until resurvey, the average number of cigarettes smoked remained about four per day. However, among those who smoked ≥10 cigarettes per day at baseline and continued to smoke until resurvey, the average number of cigarettes smoked reduced from about 15 per day at baseline to about 10 per day by resurvey. Almost all of those who were never smokers at baseline remained so at resurvey

### Smoking and all-cause mortality

**Figure 2:**
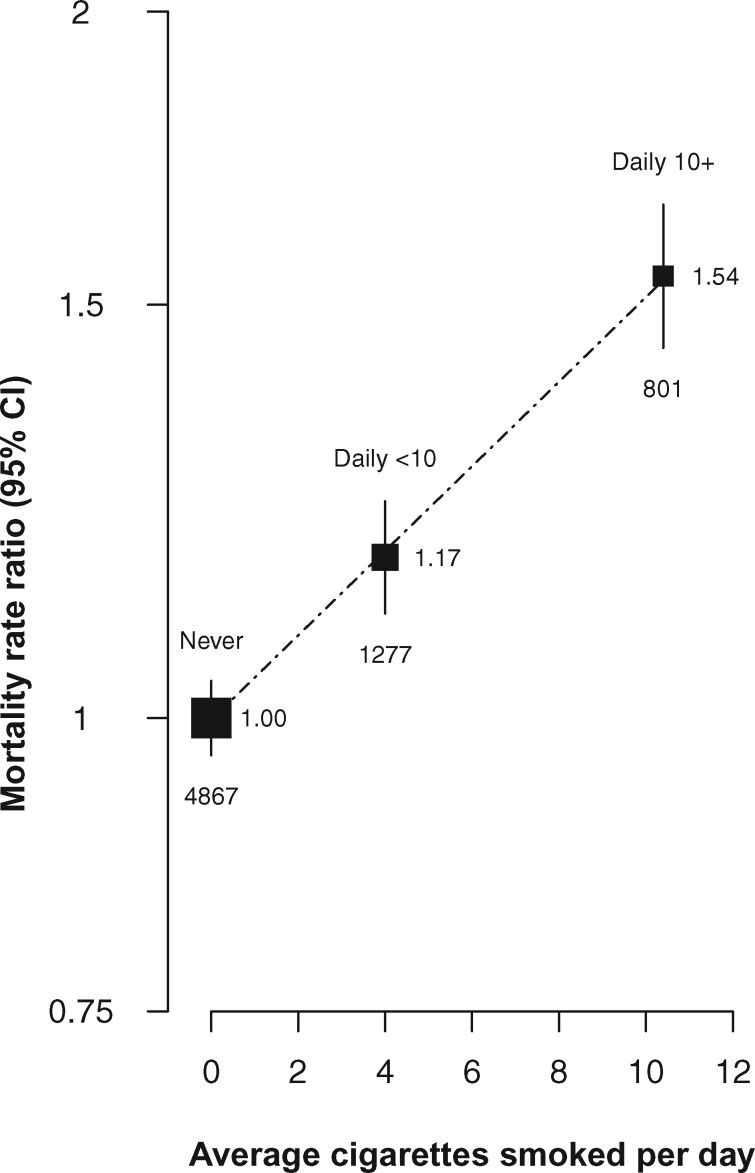
Effect of daily vs never smoking on all−cause mortality at ages 35 − 89 years, by amount smoked. RR = mortality rate ratio. Analyses exclude those with previous disease at recruitment and are adjusted for age at risk, district, highest education attained and alcohol consumption. The area of each plotting symbol is proportional to the amount of statistical information. Vertical lines through the symbols represent group-specific 95% confidence intervals, with the number of deaths in each category shown at the bottom of each line. Estimates are plotted against the mean number of cigarettes smoked per day at resurvey among participants who continued to smoke. Among non-daily smokers, the RR compared with never smokers was 0.98 (95% CI 0.90-1.07)

During follow-up, there were 10 775 deaths at ages 35–89 years among the 129 151 participants. Compared with never smokers, the RR for all-cause mortality at ages 35–89 was 0.98 (95% CI 0.90–1.07) for those who reported smoking less than daily (not plotted), 1.17 (95% CI 1.10–1.25) for daily smokers of <10 cigarettes per day at recruitment (resurvey average four cigarettes/day) and 1.54 (95% CI 1.42–1.67) for daily smokers of ≥10 cigarettes per day at recruitment (resurvey average 10 cigarettes/day) (Figure 2). The RRs associated with daily smoking were similar for men and women and for deaths at older vs younger ages (Supplementary Figure S3, available as Supplementary data at *IJE* online). Subsequent analyses of cause-specific mortality for daily versus never smokers combine men and women and deaths at different ages.

### Smoking and disease-specific mortality

**Figure 3:**
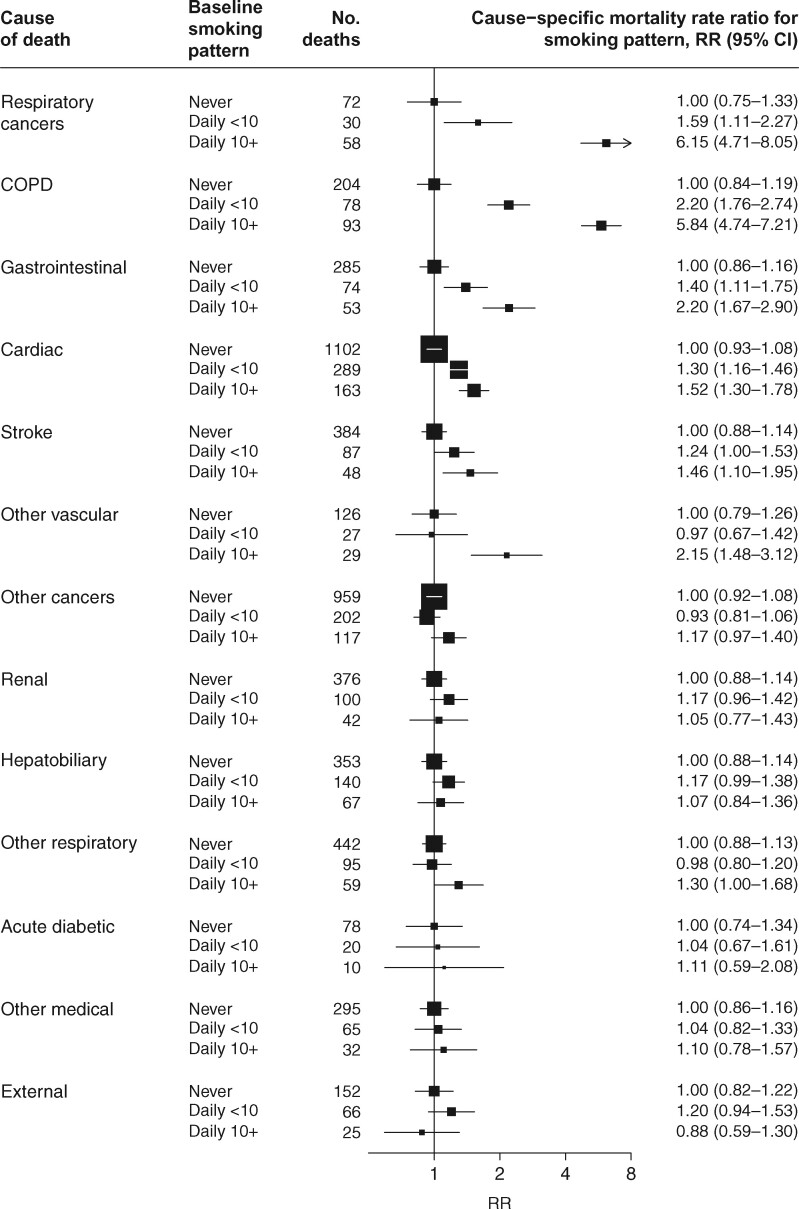
Cause−specific mortality at ages 35−89 years for daily vs never smoking. COPD = chronic obstructive pulmonary disease. RR = mortality rate ratio. Analyses exclude those with previous disease at recruitment and are adjusted for age at risk, sex, district, highest education attained and alcohol consumption. The area of each plotting symbol is proportional to the amount of statistical information, and the lines through them represent group-specific 95% confidence intervals

Clear dose-response relationships were observed between usual amount smoked and mortality risk for several causes of death, including respiratory cancer, chronic obstructive pulmonary disease (COPD), gastrointestinal diseases and vascular diseases (Figure 3). Daily smoking was little associated with other major causes of death.

### Age started smoking and smoking cessation

When daily smokers were subdivided by age started, larger RRs were generally seen for those who started smoking as children (i.e. age < 18 years) compared with those who started smoking as adults (Supplementary Figure S4, available as Supplementary data at *IJE* online). Among ever-daily smokers, those who reported having quit by recruitment had approximately half the excess mortality risk of those who reported still smoking daily when recruited; the all-cause mortality RRs compared with never smokers for these two groups were 1.15 (1.12–1.22) and 1.29 (1.25–1.37), respectively; see Supplementary Figure S5, available as Supplementary data at *IJE* online for individual causes.

### Smoking and all-cause mortality in those with previous disease

The main analyses above excluded those with previous disease at recruitment. When parallel analyses were done including the 27 940 participants with previous disease, the strength of the association of all-mortality with amount smoked was shallower in this group (as might be expected from the effects of reverse causality), but even despite this the absolute relevance of daily smoking to mortality was greater among those with than without previous disease (Supplementary Figure S6, available as Supplementary data at *IJE* online).

### Smoking and all-cause mortality by race in the USA

**Figure 4:**
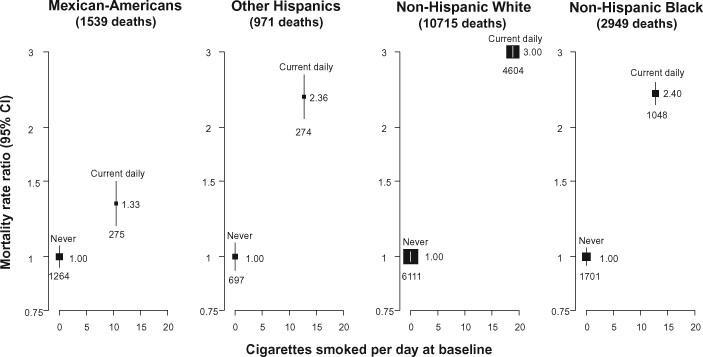
US (National Health Interview Survey) relative risk of all−cause mortality by daily vs never smoking and race, ages 30−89. RR = mortality rate ratio. Analyses exclude those with previous disease at recruitment and are adjusted for age at risk, sex, region, highest education attained and alcohol consumption. The area of each plotting symbol is proportional to the amount of statistical information. Vertical lines through the symbols represent group-specific 95% confidence intervals, with the number of deaths in each category shown at the bottom of each line (categories corresponding to former smokers are not shown). Estimates are plotted against the mean number of cigarettes smoked per day at baseline

Among 307 992 adults included in analyses of the US National Health Interview Survey, there were 29 984 (9.7%) Mexican-Americans, 21 696 (7.0%) other Hispanics, 195 837 (63.6%) non-Hispanic Whites, 42 946 (13.9%) non-Hispanic Blacks and 17 529 (5.7%) adults of other race/ethnicity. Average cigarette consumption among those who reported being daily smokers at baseline was lower for Mexican-American smokers than for smokers from other racial/ethnic groups, and the all-cause mortality RR of daily vs never smokers among Mexican-Americans was similar to that of Mexicans in the Mexico City cohort (Figure 4). In every racial/ethnic group, however, smoking was associated with substantially increased all-cause mortality compared with never smoking.

## Discussion

In this large prospective study of Mexican adults, nearly half of the men and one-fifth of the women reported being a current smoker, but many smoked less than daily or smoked only a few cigarettes per day. Low-intensity daily smoking was associated with increased mortality. Assuming the excess mortality risks associated with daily smoking are largely or wholly caused by smoking—which seems likely, given that smoking was strongly associated with causes of death known to be associated with smoking in other populations—smoking was responsible for more than one in five deaths in these Mexican smokers, and one in three deaths for those who originally reported smoking at least 10 cigarettes per day.

Smoking cessation in this population was also common, and was associated with a substantial reduction in the risks associated with continuing to smoke. Ex-daily smokers who had quit by recruitment had about half the excess mortality risk compared with those who were still smoking daily at recruitment. Proportional reductions in risk were especially pronounced for the causes of death most strongly associated with smoking, including respiratory cancers (comprising cancers of the lung and upper aerodigestive tract), COPD, gastrointestinal diseases and cardiac disease. We were unable to report the effects of smoking cessation at different ages, or for different lengths of time, due to limited information on smoking cessation at recruitment. However, the overall health benefits of quitting in this Mexican population are clear, and evidence from other global populations suggests they are likely to be greater among those who quit at a younger age (e.g. by age 40, and ideally well before 40).^3–5^^,^^16^

Smoking behaviours in Mexico are quite different to those in most other previously studied populations, with both the tendency to smoke less than daily and for daily smokers to smoke relatively few cigarettes per day. Indeed, among the 14 low- and middle-income countries that took part in the Global Adult Tobacco Surveys (GATS) in 2008–10, Mexican smokers smoked the fewest cigarettes (nine per smoker per day) of all but two countries (India and Bangladesh)—countries in which other tobacco products commonly replace cigarettes (which is not the case in Mexico).^17^ Low-intensity smoking patterns have also been documented previously among Hispanic populations in the USA^18^ and in a number of countries^7^ across Latin America. Although the current cohort includes individuals from just two districts of Mexico City recruited nearly 20 years ago, the smoking patterns seen are similar to those reported in more recent nationally representative Mexican surveys.^9^

These findings of low-intensity smoking among Mexicans led us to re-examine US data (which has previously been used)^3^ to report the association between smoking and mortality among different races. We found that the association between low-intensity smoking and all-cause mortality among Mexican-Americans in the USA was similar to the association observed in Mexico. Prospective studies are needed from other populations in which low-intensity smoking is common, to determine whether these behaviours lead to mortality risks similar to those observed in these Mexican and Mexican-American populations.

Reliable evidence on the mortality risk of low-intensity smoking is limited. In one recent US study, the estimated mortality risks associated with lifelong non-daily smoking were substantially greater than in our report^19^; indeed, they were not much lower than the risks associated with daily smoking (despite monthly cigarette consumption being more than 10 times lower among the non-daily than the daily smokers). Similarly, in another US study, the mortality risks associated with smoking <1 cigarette per day compared with 1–10 cigarettes per day were broadly similar (although very few smoked <1 cigarette per day).^20^ However, neither of these studies was able to assess the impact of possible changes in smoking behaviour over time, and so it is possible that the smoking exposure groups studied were subject to substantial misclassification biases during the period of follow-up. In other large studies of smoking and mortality, including studies in Cuba^21^ and the UK^5^ in addition to this Mexican study, resurvey values have helped quantify and therefore correct for this source of bias.

A particular strength of the current study is its size, which enabled the reliable estimation of the hazards of low-intensity smoking in this population. A further strength is that whereas participants were categorized according to their baseline smoking status, the availability of a resurvey of survivors in 2015–19 allowed the RRs associated with those baseline-defined groups to be interpreted according to the estimated usual number of cigarettes smoked per day during the study. Sufficiently detailed information was sought to separate ‘current’ (and ex-) smokers into those who smoked daily and those who smoked less than daily, which is an important distinction in this population in which non-daily smoking is common, and may not lead to persistent smoking (and its adverse health effects). However, information about smoking cessation was not well characterized, and we could not assess the benefits of smoking cessation at different ages or for different durations. We also did not collect information on the type of cigarette smoked, but previous studies have indicated that, at least with respect to nicotine intake, the number of cigarettes smoked is more relevant than the type (e.g. ‘regular’ or ‘light’ cigarettes).^22^^,^^23^ Finally, because we excluded those with diabetes or other chronic diseases at recruitment (in order to reduce the extent of reverse causality bias), the included participants would have been somewhat healthier on average than the general population.

In summary, the risk of death associated with smoking in this Mexican population was lower than in other, particularly high-income, populations, but still accounted for significant excess mortality among daily smokers. Similar associations were observed among Mexican-Americans in the USA. This study confirms that even low levels of smoking are harmful, and that those who quit can avoid much of the excess risk of death that would otherwise be caused by continued smoking.

## Supplementary data

Supplementary data are available at *IJE* online.

## Data availability

We welcome requests from researchers who wish to access data from the Mexico City Prospective Study. If you are interested in obtaining data from the study for research purposes, or in collaborating with us on a specific research proposal, please visit our study website [https://www.ctsu.ox.ac.uk/research/prospective-blood-based-study-of-150-000-individuals-in-mexico] where you can download our Data and Sample Access Policy in either English or Spanish.

## Funding

The Mexico City Prospective Study has received funding from the Mexican Health Ministry, the National Council of Science and Technology for Mexico, the Wellcome Trust, Cancer Research UK, British Heart Foundation (BHF) and the UK Medical Research Council. D.A.R acknowledges support from the BHF Centre for Research Excellence, Oxford (grant code RE/13/1/30181). W.G.H. is supported by an MRC-Kidney Research UK Professor David Kerr Clinician Scientist Award. The funding sources had no role in the design, conduct or analysis of the study or the decision to submit the manuscript for publication.

## Author contributions

J.A-D., R.C., P.K-M, R.P. and R.T-C. established the cohort. J.A-D., P.K-M., R.R-R. and R.T-C. gathered the data. W.G.H., R.R-R. and R.W. linked, reviewed and/or adjudicated the death certificates. B.T. developed the study design, did the analyses and wrote the first draft of the paper under the supervision of J.E., B.L., S.L. and R.P. All authors contributed to revision of the report and agreed to its publication. J.A-D. and J.R.E. had full access to all the data in the study and take responsibility for the integrity of the data and the accuracy of the analysis.

## Conflict of interest

R.C. is a British Heart Foundation Chair-holder, and reports personal fees from UK Biobank, grants from Merck & Co, grants from Medicines Company (now Novartis) and other from Pfizer, outside the submitted work; in addition, R.C. has a patent for a statin-related myopathy genetic test licensed to University of Oxford from Boston Heart Diagnostics (R.C. has waived any personal reward). J.R.E. and W.G.H. report grants from Boehringer Ingelheim, outside the submitted work. S.L. declares research funding from the US Centers for Disease Control and Prevention Foundation (with support from Amgen), outside the submitted work. All other authors declare no conflicts.

## Supplementary Material

dyab013_Supplementary_Data
