# Supplementary material for: Low-intensity daily smoking and cause-specific mortality in Mexico: prospective study of 150 000 adults
Source: Int J Epidemiol. 2021 Feb 28;50(3):955–64. doi: 10.1093/ije/dyab013 (PMC8271211; doi:10.1093/ije/dyab013)
Supplement: dyab013_Supplementary_Data [file dyab013_supplementary_data.pdf]

Supplementary Material

Low-intensity daily smoking and cause-specific mortality in Mexico: prospective study of 150 000 adults

Figures **Pg**

S1. Current, ex and ever smoking, by year of birth	2
S2. Amount smoked and age started, by year of birth	3
S3. All−cause mortality for daily vs never smoking, by age at risk, sex and amount smoked	4
S4. Cause−specific mortality at ages 35−89 years for daily versus never smoking, by age started smoking	5
S5. Cause−specific mortality at ages 35−89 years for daily versus ex−daily versus never smoking	6
S6. Effect of daily vs never smoking on all−cause mortality at ages 35−89 years, by amount smoked and history of prior disease	7

Tables

S1. Numbers of deaths at ages 35-89 years by underlying cause (ICD-10 code)	8
S2. Characteristics of 14,565 male and 14,751 female ex-smokers aged 35-89 at recruitment, by previous smoking habit	10
S3. Changes in reported smoking habits from baseline to resurvey	11

Brief report on analyses of the 1997-2014 US National Health Interview Surveys	12

Figure S1: Current, ex and ever smoking, by year of birth

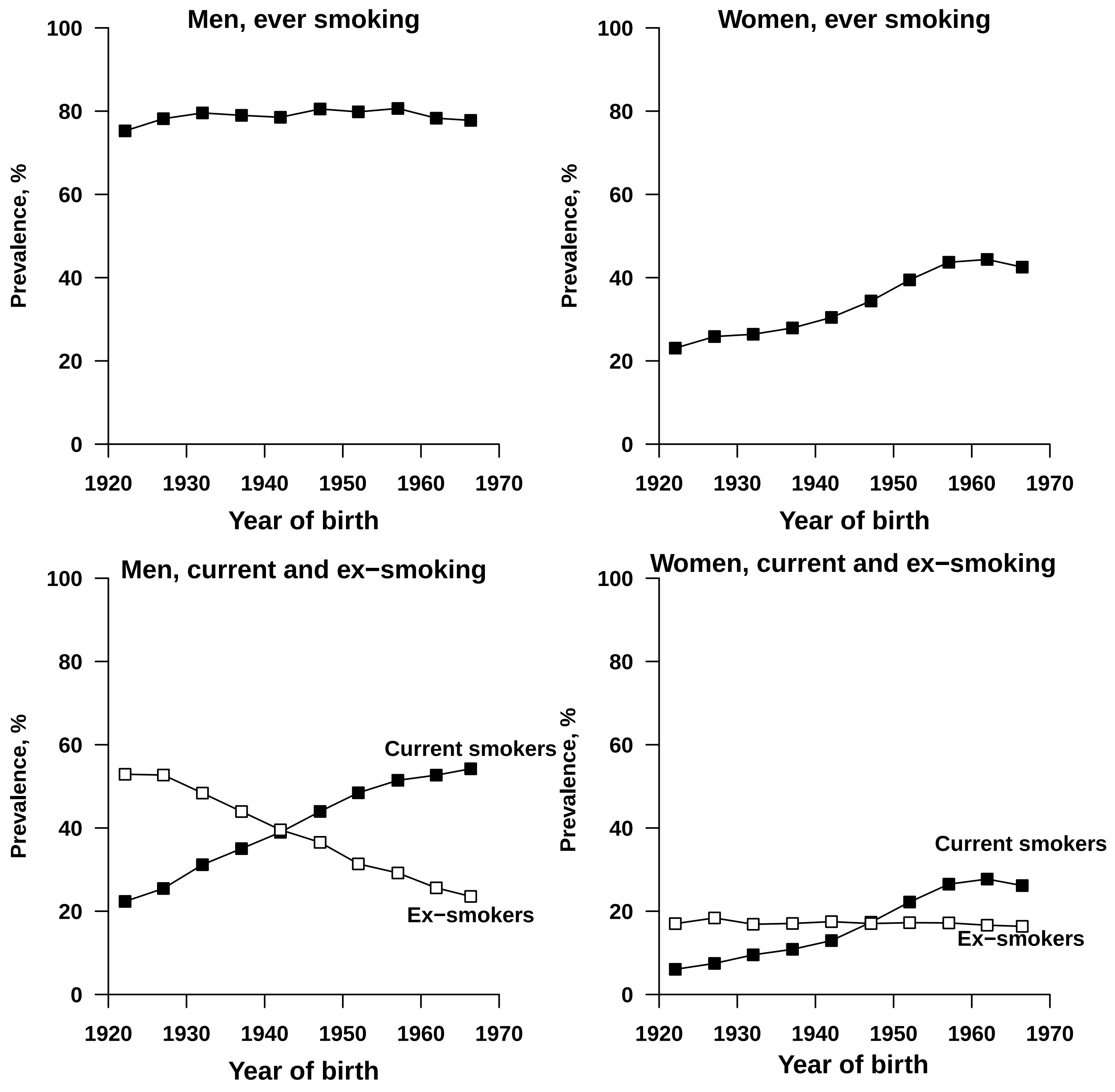

Figure S2: Amount smoked and age started, by year of birth

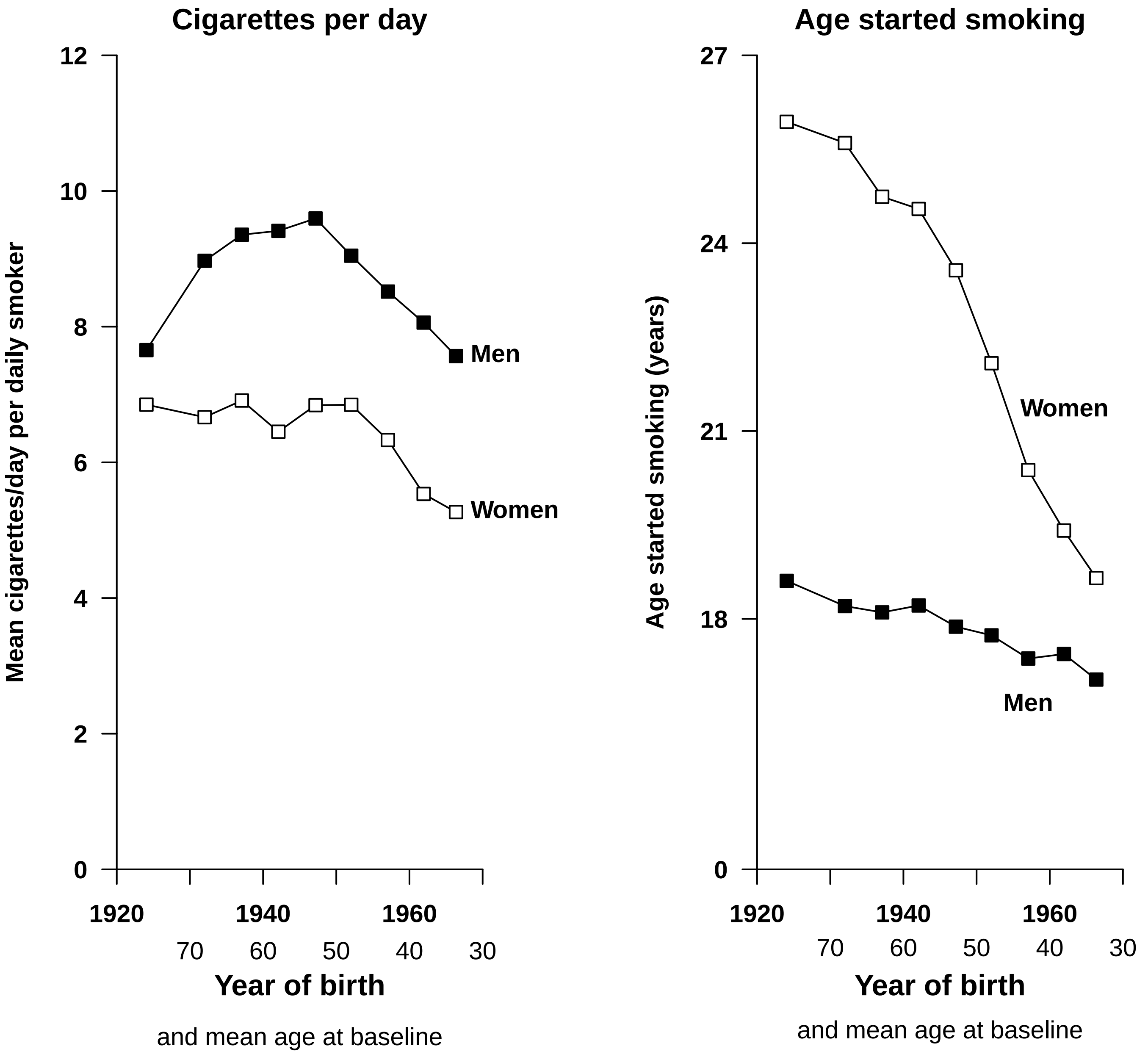

Figure S3: All−cause mortality for daily vs never smoking, by age at risk, sex and amount smoked

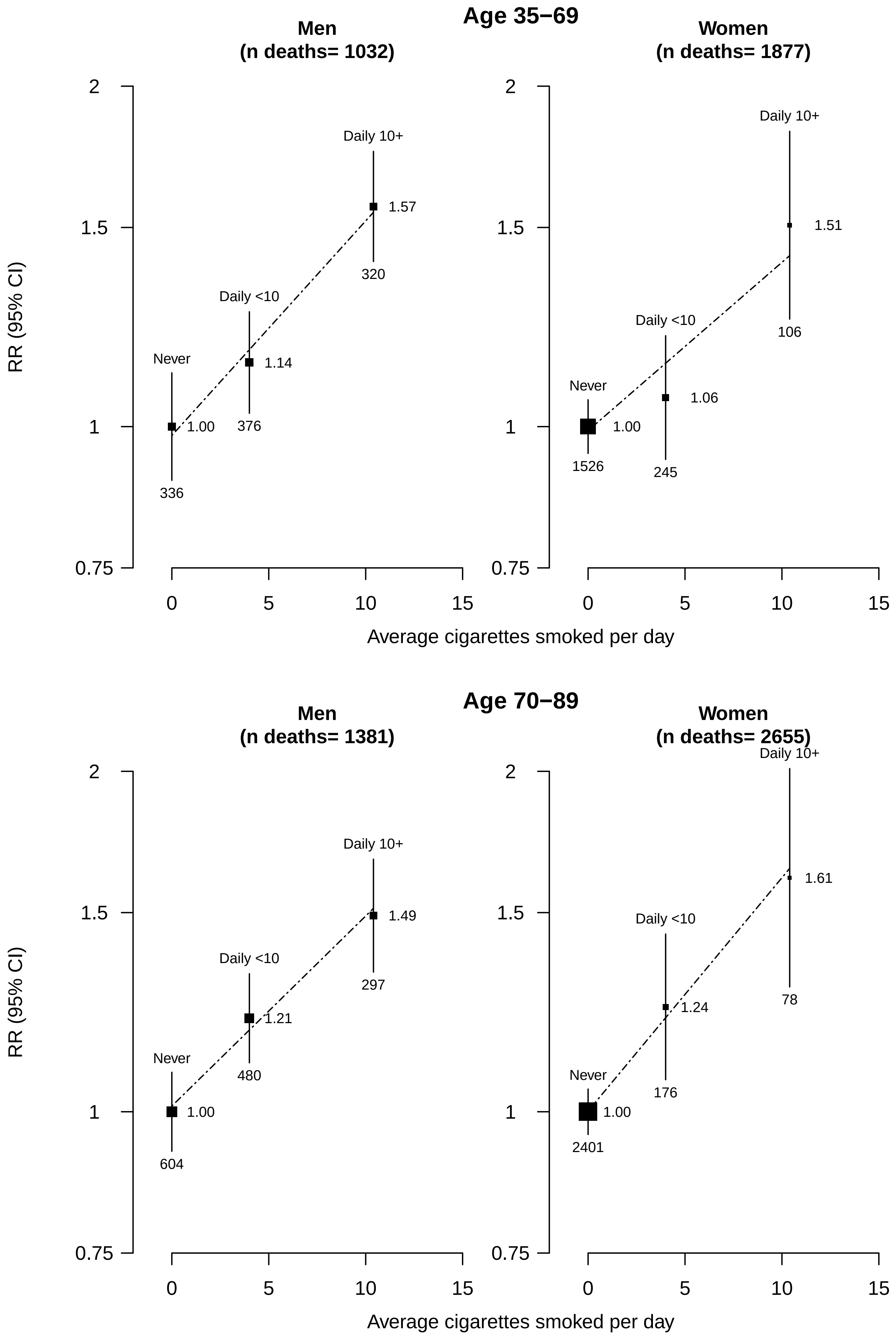

*Excludes those with baseline chronic disease, and adjusted for age at risk, district, education, and alcohol consumption.

Figure S4: Cause−specific mortality at ages 35−89 years for daily versus never smoking, by age started smoking

Cause of death	Baseline smoking pattern*	No. deaths	Cause−specific mortality rate ratio for smoking pattern, RR (95% CI)
Respiratory cancers	Never	72	1.00 (0.75–1.33)
	Age 18+	30	2.12 (1.49–3.03)
	Age <18	58	4.01 (3.06–5.26)
COPD	Never	204	1.00 (0.84–1.19)
	Age 18+	79	3.15 (2.53–3.93)
	Age <18	92	3.38 (2.73–4.18)
Gastrointestinal	Never	285	1.00 (0.86–1.16)
	Age 18+	58	1.50 (1.16–1.94)
	Age <18	69	1.79 (1.40–2.28)
Cardiac	Never	1102	1.00 (0.93–1.08)
	Age 18+	215	1.31 (1.15–1.50)
	Age <18	237	1.43 (1.26–1.63)
Stroke	Never	384	1.00 (0.88–1.14)
	Age 18+	69	1.32 (1.04–1.67)
	Age <18	66	1.29 (1.01–1.65)
Other vascular	Never	126	1.00 (0.79–1.26)
	Age 18+	21	1.04 (0.68–1.59)
	Age <18	35	1.67 (1.18–2.35)
Other cancers	Never	959	1.00 (0.92–1.08)
	Age 18+	179	1.07 (0.92–1.24)
	Age <18	140	0.92 (0.77–1.09)
Renal	Never	376	1.00 (0.88–1.14)
	Age 18+	76	1.24 (0.99–1.55)
	Age <18	66	1.02 (0.80–1.31)
Hepatobiliary	Never	353	1.00 (0.88–1.14)
	Age 18+	92	1.10 (0.89–1.34)
	Age <18	115	1.17 (0.97–1.41)
Other respiratory	Never	442	1.00 (0.88–1.13)
	Age 18+	82	1.17 (0.94–1.45)
	Age <18	72	0.98 (0.77–1.24)
Acute diabetic	Never	78	1.00 (0.74–1.34)
	Age 18+	14	1.05 (0.62–1.77)
	Age <18	16	1.07 (0.65–1.77)
Other medical	Never	295	1.00 (0.86–1.16)
	Age 18+	38	0.84 (0.61–1.16)
	Age <18	59	1.29 (1.00–1.68)
External	Never	152	1.00 (0.82–1.22)
	Age 18+	37	0.99 (0.72–1.37)
	Age <18	54	1.19 (0.90–1.56)

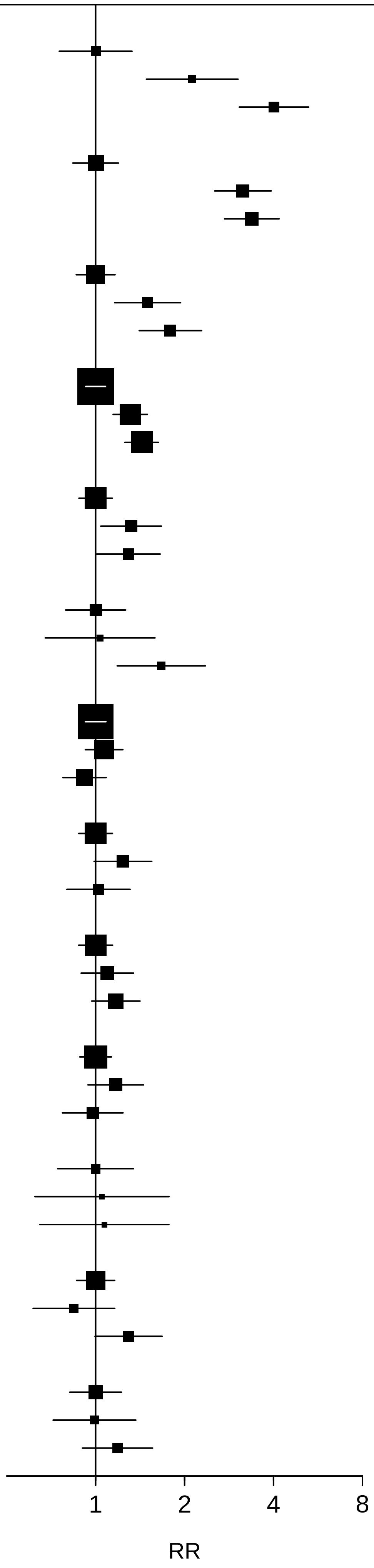

CI=Confidence interval; RR=Rate ratio. *Risks associated with starting at age 24 years, or starting at age 14 years on average, respectively. Adjustments and exclusions as for RRs in Figure 1. Ill−defined deaths not pictured; starting age<18 years associated with RR of 0.77 (95% CI 0.34−1.75).

Figure S5: Cause−specific mortality at ages 35−89 years for daily versus ex−daily versus never smoking

Cause of death	Baseline smoking pattern*	No. deaths	Cause−specific mortality rate ratio for smoking pattern, RR (95% CI)
Respiratory cancers	Never	72	1.00 (0.76–1.32)
	Ex−daily	48	1.43 (1.07–1.90)
	Daily	88	3.02 (2.43–3.74)
COPD	Never	204	1.00 (0.84–1.19)
	Ex−daily	132	1.59 (1.34–1.89)
	Daily	171	3.28 (2.81–3.83)
Gastrointestinal	Never	285	1.00 (0.86–1.16)
	Ex−daily	126	1.18 (0.99–1.41)
	Daily	127	1.63 (1.37–1.95)
Cardiac	Never	1102	1.00 (0.93–1.08)
	Ex−daily	546	1.25 (1.15–1.36)
	Daily	452	1.37 (1.25–1.51)
Stroke	Never	384	1.00 (0.88–1.14)
	Ex−daily	194	1.42 (1.23–1.64)
	Daily	135	1.31 (1.10–1.56)
Other vascular	Never	126	1.00 (0.79–1.26)
	Ex−daily	64	1.23 (0.96–1.58)
	Daily	56	1.35 (1.03–1.76)
Other cancers	Never	959	1.00 (0.92–1.08)
	Ex−daily	340	0.98 (0.88–1.09)
	Daily	319	1.00 (0.90–1.12)
Renal	Never	376	1.00 (0.88–1.14)
	Ex−daily	149	0.97 (0.82–1.15)
	Daily	142	1.13 (0.96–1.34)
Hepatobiliary	Never	353	1.00 (0.88–1.14)
	Ex−daily	192	1.16 (1.01–1.34)
	Daily	207	1.13 (0.99–1.30)
Other respiratory	Never	444	1.00 (0.88–1.13)
	Ex−daily	180	0.98 (0.85–1.14)
	Daily	154	1.07 (0.91–1.26)
Acute diabetic	Never	78	1.00 (0.74–1.34)
	Ex−daily	29	0.92 (0.63–1.34)
	Daily	30	1.06 (0.73–1.52)
Other medical	Never	295	1.00 (0.86–1.16)
	Ex−daily	133	1.22 (1.03–1.46)
	Daily	97	1.06 (0.87–1.30)
External	Never	152	1.00 (0.82–1.22)
	Ex−daily	76	0.98 (0.78–1.23)
	Daily	91	1.09 (0.88–1.35)

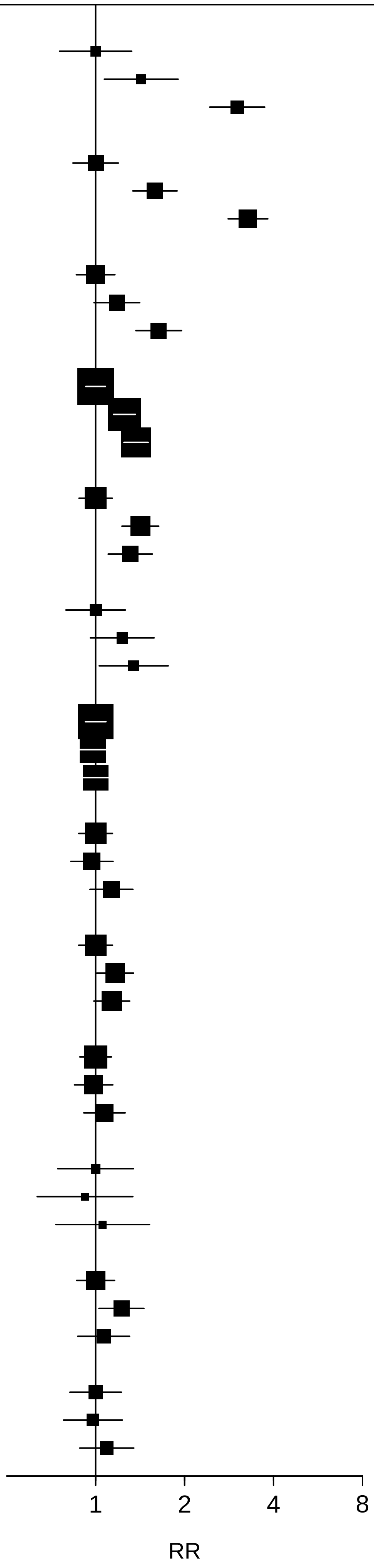

CI=Confidence interval; RR=Rate ratio. *Risks associated with smoking 0, 4, and 16 cigarettes per day on average at recruitment, respectively. Adjustments and exclusions as for RRs in Figure 2.

Figure S6: Effect of daily vs never smoking on all−cause mortality at ages 35−89 years, by amount smoked and history of prior chronic disease

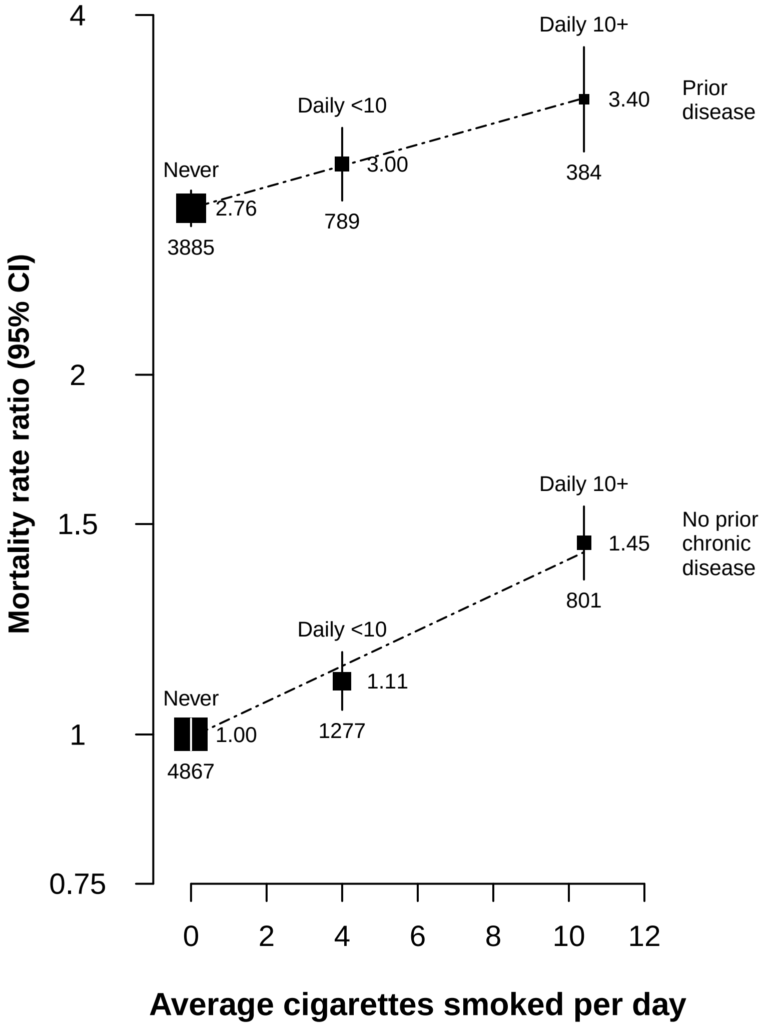

Analyses as Figure 2, except that participants with prior chronic disease are now included. RR estimates among those without prior disease differ slightly from those in Figure 2 because of minor differences in the impact of adjusting for confounders in the two regression models.

Table S1: Numbers of deaths at ages 35-89 years by underlying cause (ICD-10 code)

Underlying cause of death	ICD-10 codes (and number of deaths)
Cardiac (n=2485)	I050 (2), I051 (2), I059 (15), I070 (1), I071 (3), I080 (5), I081 (1), I091 (1), I099 (17), I110 (131), I119 (22), I200 (2), I209 (4), I210 (6), I211 (10), I219 (1833), I221 (2), I229 (1), I248 (2), I249 (9), I251 (51), I255 (3), I258 (4), I259 (78), I270 (8), I272 (3), I279 (14), I289 (1), I301 (1), I309 (1), I318 (1), I330 (3), I340 (6), I348 (1), I350 (15), I351 (1), I358 (2), I359 (1), I369 (1), I38X (4), I420 (13), I426 (1), I429 (2), I441 (1), I442 (4), I443 (1), I469 (3), I471 (1), I472 (2), I489 (6), I48X (11), I490 (6), I499 (7), I500 (53), I501 (13), I509 (69), I518 (2), I519 (8), Q210 (1), Q231 (1), Q238 (1), Q248 (2), R570 (9)
Stroke (n=835)	F019 (5), I600 (2), I602 (1), I608 (1), I609 (69), I610 (1), I615 (1), I618 (1), I619 (218), I620 (8), I629 (5), I633 (3), I634 (17), I635 (18), I638 (1), I639 (80), I64X (108), I652 (1), I671 (12), I672 (2), I673 (1), I674 (3), I678 (94), I679 (132), I690 (1), I691 (1), I693 (7), I694 (8), I698 (34)
Other vascular (n=271)	E115 (4), E145 (7), I260 (4), I269 (71), I709 (2), I710 (5), I712 (1), I713 (6), I714 (5), I718 (1), I719 (2), I729 (1), I739 (4), I741 (2), I771 (11), I776 (1), I802 (7), I803 (3), I822 (1), I828 (2), I829 (4), I830 (1), I872 (9), I879 (1), I888 (1), I99X (2), K550 (94), K551 (1), K552 (2), K559 (15), K761 (1)
Respiratory cancers (n=229)	C009 (1), C01X (1), C029 (10), C049 (1), C050 (1), C059 (1), C069 (3), C07X (5), C089 (1), C109 (4), C119 (3), C139 (1), C140 (5), C329 (17), C33X (2), C340 (1), C349 (163), D380 (1), D381 (6), D383 (2)
Other cancers (n=1906)	C159 (16), C160 (3), C169 (194), C170 (11), C179 (1), C181 (1), C187 (3), C189 (118), C19X (8), C20X (20), C210 (1), C220 (47), C221 (22), C227 (1), C229 (90), C23X (28), C240 (9), C241 (9), C248 (3), C249 (19), C250 (25), C259 (113), C260 (2), C269 (1), C310 (1), C319 (1), C37X (1), C383 (1), C384 (1), C402 (2), C410 (1), C412 (3), C414 (1), C419 (6), C437 (1), C438 (1), C439 (16), C442 (1), C444 (1), C445 (1), C447 (1), C449 (8), C450 (1), C451 (1), C459 (4), C469 (1), C479 (1), C480 (11), C482 (7), C490 (1), C492 (3), C493 (1), C499 (14), C509 (170), C519 (3), C539 (92), C541 (15), C549 (2), C55X (11), C56X (91), C609 (2), C61X (126), C629 (4), C64X (75), C65X (1), C679 (27), C680 (3), C709 (2), C710 (17), C711 (1), C718 (3), C719 (39), C729 (1), C73X (27), C749 (1), C751 (2), C753 (1), C759 (3), C760 (5), C762 (6), C763 (3), C764 (1), C765 (1), C779 (1), C780 (4), C786 (2), C787 (14), C788 (3), C793 (3), C794 (2), C795 (1), C796 (1), C798 (3), C800 (34), C809 (14), C80X (1), C811 (1), C817 (1), C819 (10), C829 (1), C833 (11), C839 (1), C844 (1), C845 (2), C851 (1), C857 (1), C859 (45), C880 (1), C900 (43), C902 (1), C910 (24), C911 (2), C919 (2), C920 (29), C921 (6), C924 (1), C927 (3), C929 (2), C930 (1), C950 (2), C959 (5), C969 (1), C97X (2), D371 (2), D372 (1), D374 (3), D376 (8), D377 (8), D391 (3), D410 (1), D430 (10), D431 (2), D432 (1), D449 (1), D483 (1), D484 (1), D486 (1), D487 (6), D489 (1)
COPD (n=568)	J40X (3), J42X (23), J439 (52), J440 (219), J441 (3), J448 (12), J449 (256)
Other respiratory (n=921)	A150 (1), A162 (10), A169 (1), B206 (2), B909 (1), E840 (1), E848 (1), J09 (2), J100 (2), J150 (1), J151 (2), J152 (1), J157 (1), J159 (26), J180 (79), J181 (43), J182 (1), J188 (1), J189 (493), J209 (4), J22X (23), J348 (1), J459 (18), J46X (4), J47X (3), J64X (9), J65X (1), J679 (2), J680 (1), J684 (1), J80X (3), J81X (10), J841 (104), J849 (8), J852 (1), J860 (1), J869 (5), J90X (3), J939 (1), J949 (1), J960 (2), J961 (2), J969 (3), J980 (1), J981 (1), J984 (22), J985 (3), J988 (13), Q311 (1)
Gastrointestinal (n=636)	A047 (3), A090 (17), A099 (40), A09X (12), A183 (1), K052 (1), K088 (1), K102 (1), K137 (1), K20X (2), K222 (1), K223 (3), K228 (2), K251 (4), K254 (21), K255 (6), K256 (1), K259 (11), K264 (16), K265 (5), K269 (4), K272 (1), K274 (3), K275 (2), K290 (12), K291 (3), K292 (1), K295 (7), K296 (1), K297 (8), K311 (1), K316 (1), K318 (9), K352 (5), K353 (4), K358 (2), K37X (3), K389 (1), K403 (6), K404 (2), K409 (2), K413 (3), K420 (5), K421 (1), K430 (3), K439 (2), K460 (3), K461 (1), K469 (5), K513 (1), K519 (2), K560 (3), K562 (6), K566 (52), K572 (2), K573 (4), K578 (10), K579 (17), K593 (5), K610 (3), K611 (1), K628 (1), K631 (21), K632 (5), K635 (1), K638 (1), K639 (7), K650 (8), K658 (1), K659 (61), K669 (1), K918 (1), K920 (16), K921 (1), K922 (154), K931 (1)

Table S1: Numbers of deaths at ages 35-89 years by underlying cause (ICD-10 code)

Underlying cause of death	ICD-10 codes (and number of deaths)
Renal (n=830)	E102 (3), E112 (151), E142 (63), I120 (139), I129 (3), I130 (1), I131 (3), I132 (13), N002 (1), N009 (6), N039 (12), N059 (3), N12X (5), N133 (1), N151 (9), N170 (2), N179 (90), N180 (11), N185 (6), N189 (125), N19X (24), N200 (6), N201 (1), N202 (1), N289 (2), N308 (1), N390 (145), Q612 (1), Q613 (1), Q619 (1)
Hepatobiliary (n=932)	B162 (1), B169 (2), B171 (21), B181 (2), B182 (11), B189 (1), B190 (2), D134 (1), I850 (14), I859 (4), K701 (20), K702 (1), K703 (142), K704 (13), K709 (16), K711 (1), K716 (1), K720 (6), K721 (50), K729 (164), K739 (2), K740 (1), K742 (1), K743 (3), K744 (1), K745 (2), K746 (249), K750 (8), K754 (2), K760 (1), K766 (10), K767 (11), K769 (16), K800 (6), K801 (6), K802 (2), K803 (6), K805 (2), K810 (21), K811 (8), K819 (8), K820 (3), K821 (2), K822 (1), K828 (1), K829 (6), K830 (17), K831 (2), K851 (1), K852 (3), K858 (3), K859 (30), K85X (21), K861 (1), K863 (1), Q447 (1)
Acute diabetic (n=163)	E100 (1), E110 (42), E111 (44), E140 (36), E141 (38), E162 (2)
Other medical (n=643)	A181 (1), A182 (1), A188 (1), A199 (3), A415 (1), A418 (1), A419 (137), A810 (2), A86X (1), B200 (1), B201 (1), B207 (2), B208 (8), B210 (1), B212 (1), B227 (1), B238 (4), B24X (5), B690 (2), B699 (1), D033 (1), D181 (1), D27X (1), D329 (8), D361 (1), D369 (1), D464 (1), D469 (8), D479 (1), D530 (1), D589 (1), D594 (1), D619 (8), D649 (2), D65X (1), D682 (1), D691 (1), D693 (2), D694 (1), D695 (1), D696 (1), D699 (3), D70X (1), D733 (1), D762 (1), E035 (1), E039 (10), E049 (1), E055 (1), E059 (3), E065 (1), E119 (5), E146 (2), E147 (1), E149 (3), E230 (1), E249 (1), E279 (1), E43X (5), E46X (6), E500 (1), E660 (1), E835 (1), E871 (1), E875 (1), E878 (5), E889 (1), F03X (17), F09X (1), F102 (5), F182 (1), F329 (3), G009 (3), G039 (2), G049 (5), G10X (2), G121 (1), G122 (16), G20X (22), G219 (4), G301 (3), G309 (13), G319 (1), G35X (2), G379 (1), G409 (15), G419 (1), G439 (1), G589 (1), G610 (2), G629 (1), G700 (1), G710 (3), G809 (1), G819 (1), G822 (1), G919 (4), G931 (9), G934 (3), G935 (1), G939 (1), G958 (1), G959 (1), L020 (1), L021 (3), L022 (2), L023 (1), L031 (5), L032 (1), L038 (1), L039 (4), L088 (1), L089 (18), L100 (2), L108 (1), L109 (1), L512 (3), L899 (13), L89X (3), L905 (5), L921 (1), L984 (12), L988 (1), L989 (1), M009 (2), M050 (2), M068 (1), M069 (29), M100 (1), M109 (1), M165 (1), M189 (1), M199 (2), M300 (1), M311 (1), M313 (1), M319 (1), M321 (4), M329 (1), M348 (1), M349 (1), M350 (2), M471 (1), M600 (1), M623 (13), M726 (6), M798 (22), M809 (2), M819 (4), M869 (1), N40X (16), N410 (1), N498 (4), N499 (1), N719 (4), N739 (3), N823 (1), N948 (1), O720 (1)
External (n=398)	S068 (1), S720 (2), T782 (1), V011 (1), V031 (2), V049 (1), V051 (1), V059 (1), V093 (3), V099 (82), V149 (1), V209 (1), V299 (1), V439 (1), V494 (1), V499 (11), V580 (1), V581 (1), V719 (1), V785 (1), V799 (1), V878 (6), V892 (7), V899 (5), W018 (1), W040 (1), W068 (1), W100 (13), W104 (2), W105 (1), W108 (2), W126 (1), W130 (16), W134 (3), W135 (1), W138 (1), W139 (2), W170 (4), W172 (1), W174 (2), W176 (1), W178 (1), W179 (1), W180 (8), W181 (1), W184 (4), W188 (1), W189 (1), W190 (16), W194 (2), W195 (1), W199 (4), W206 (1), W228 (1), W250 (1), W314 (1), W340 (1), W370 (1), W557 (1), W744 (1), W748 (2), W780 (2), W784 (1), W789 (1), W808 (1), W849 (2), W87 (1), W871 (1), W878 (1), X09 (1), X090 (3), X094 (1), X449 (1), X470 (1), X53 (1), X530 (2), X590 (4), X594 (1), X598 (1), X599 (28), X650 (1), X680 (1), X700 (8), X702 (1), X708 (1), X740 (5), X780 (1), X910 (2), X914 (2), X950 (4), X954 (15), X955 (1), X959 (1), X990 (8), X994 (4), X999 (1), Y018 (1), Y040 (1), Y044 (2), Y084 (1), Y090 (2), Y099 (1), Y159 (1), Y200 (1), Y239 (1), Y240 (2), Y249 (1), Y260 (1), Y265 (1), Y300 (1), Y330 (1), Y334 (1), Y338 (1), Y340 (4), Y344 (6), Y345 (1), Y349 (12), Y579 (2), Y609 (1), Y839 (2), Y86X (2), Y899 (1)

Table S2: Characteristics of 14,565 male and 14,751 female ex-smokers aged 35-89 at recruitment, by previous smoking habit

	**Male ex-smokers (*n*=14,565)**		**Female ex-smokers (*n*=14,751)**	
	**Previously smoked less than daily (*n*=4716)**	**Previously smoked daily (*n*=9849)**	**Previously smoked less than daily (*n*=6746)**	**Previously smoked daily (*n*=8005)**
**Smoking behaviour**				
Age started smoking (years)	18.5 (5.9)	17.2 (5.6)	22.4 (8.1)	21.6 (8.4)
Quit within the past 3 years	15%	17%	16%	19%
Others smoke inside home	16%	20%	33%	33%
**Age (years)**	52 (13)	56 (14)	48 (11)	52 (13)
**Resident in Iztapalapa**	57%	55%	59%	56%
**Education attained**				
University	28%	22%	18%	16%
High school	25%	23%	31%	26%
Elementary school	39%	44%	43%	45%
Other	8%	12%	8%	13%
**Drinking behaviour**				
Drink at least weekly	49%	45%	61%	54%
Drink less than weekly	37%	42%	19%	24%
Former drinker	9%	8%	6%	5%
Never drinker	5%	5%	15%	17%
**Anthropometry and blood pressure**				
BMI (kg/m^2)	28.2 (4.0)	28.3 (4.3)	29.6 (5.2)	29.9 (5.5)
Waist-to-hip ratio	0.95 (0.07)	0.96 (0.06)	0.87 (0.07)	0.88 (0.07)
Systolic BP (mmHg)	128 (15)	130 (16)	124 (16)	126 (17)
Diastolic BP (mmHg)	84 (10)	85 (10)	81 (10)	83 (10)

Mean (SD) or percent shown.

Table S3: Changes in reported smoking habits from baseline to resurvey

	Baseline-defined smoking status				
			Current smoker (*n*=2328)		
	Never smoker (*n*=4659)	Ex-smoker (*n*=1766)	Less than daily (*n*=896)	Daily <10/day (*n*=1045)	Daily ≥10/day (*n*=387)
**Smoking status at resurvey**					
Never smoker	4264 (92%)	465 (26%)	172 (19%)	54 (5%)	8 (2%)
Ex smoker (not for illness)	292 (6%)	936 (53%)	407 (45%)	289 (28%)	52 (13%)
Ex smoker (for illness)	25 (1%)	220 (12%)	67 (7%)	147 (14%)	49 (13%)
Smoke < daily	35 (1%)	59 (3%)	156 (17%)	127 (12%)	14 (4%)
Smoke < 10 cigs/day	10 (<0.5%)	45 (3%)	66 (7%)	354 (34%)	107 (28%)
Smoke 10+ cigs/day	1 (<0.5%)	6 (<0.5%)	3 (<0.5%)	56 (5%)	155 (40%)
Incomplete information*	32 (1%)	35 (2%)	25 (3%)	18 (2%)	2 (1%)
**Average daily cigs**					
At baseline	-	-	0.1 (0.3)	3.8 (2.1)	15.5 (7.2)
At resurvey, per smoker †	1.8 (4.4)	2.7 (3.9)	1.3 (2.0)	4.0 (3.8)	10.5 (6.9)
At resurvey, all participants ‡	0.0 (0.5)	0.2 (1.2)	0.3 (1.1)	2.1 (3.4)	7.5 (7.5)
**Inhale smoke**					
At baseline	-	-	517 (58%)	758 (73%)	315 (81%)
At resurvey	27 (1%)	81 (5%)	159 (18%)	391 (37%)	204 (53%)
**Ever tried to quit §**					
At baseline	-	-	329 (37%)	482 (46%)	149 (39%)
At resurvey	30 (1%)	84 (5%)	168 (19%)	397 (38%)	208 (54%)

n (column %) or mean (SD) shown. * Where information given is insufficient to categorise exposure (e.g. when participant reports ex-smoking but not years since quitting, or current smoking but not frequency of smoking). † Among those who reported to be a current smoker at resurvey. ‡ Among all participants in the original baseline-defined group (assigning zero cigarettes to those reporting never or ex smoking at resurvey). § At the baseline assessment, only current smokers were asked about quitting behaviours.

Brief report on analyses of the 1997-2014 US National Health Interview Surveys

Background: We investigate the association between daily versus never smoking and all-cause mortality by race in the United States, with a focus on Mexican-Americans versus other groups.

Methods: Data were pooled from the 1997-2014 waves of the US National Health Interview Survey, a population-based, cross-sectional health survey conducted annually among non-institutionalised civilians across the United States. Households were randomly selected using a stratified, multistage design, with one adult from each household selected to answer questions about health status, health behaviours, and access to healthcare. Between 1997-2014, 231 707 women and 182 773 men aged 30-84 and eligible for mortality linkage (>95% of participants interviewed) were recruited into the study. All-cause mortality was tracked to 1 January 2016 through probabilistic linkage to the National Death Index by the National Center for Health Statistics, using a combination of personal information (including name, Social Security number, and birth date) as provided at recruitment.

Statistical analyses were conducted in such a way as to maximise comparability with the main analyses among Mexicans, with a few minor differences. For these US analyses, participants aged ≥85 years at recruitment were excluded (as all participants aged >84 were assigned age 85 years, leaving their true age at recruitment unknown), and, because of limited power for race-specific analyses, comparisons were restricted to daily versus never smokers without further stratification. Analyses adjusted for age at risk (in 5-year age groups), sex, region (4 groups), alcohol consumption (5 groups), and education (3 groups). Unlike in the Mexican analyses, RRs were plotted at the mean number of cigarettes smoked per day at recruitment (as no resurvey was done).

Findings: Of the 414 480 participants aged 30-84 years when enrolled in the US NHIS, 10 513 (%) had with missing information on smoking or relevant covariates and 95 975 (%) with a history of major chronic disease were excluded. The remaining 307 992 participants included 29 984 (9.7%) Mexican-Americans, 21 696 (7.0%) other Hispanics, 195 837 (63.6%) non-Hispanic Whites, 42 946 (13.9%) non-Hispanic Blacks, and 17 529 (5.7%) people of other races.

Overall, there were 16 698 deaths at ages 30-89 years during 2.2 million person-years of follow-up among never or daily smokers at recruitment. Average cigarette consumption among those who reported being daily smokers at recruitment was lower for Mexican-American smokers than for smokers from other ethnicity groups (Figure 4). Compared with never smoking, the all-cause mortality RRs associated with daily smoking at recruitment were 1.33 (95% CI 1.17-1.52) among Mexican-Americans, 2.36 (2.07-2.69) among other Hispanics, 3.00 (2.80-3.21) among non-Hispanic Whites, 2.40 (2.20-2.61) among non-Hispanic Blacks, and 2.04 (1.74-2.39) among those of other race/ethnicity.

Interpretation: In every racial/ethnic group, daily smoking at recruitment was associated with substantial increases in all-cause mortality. Similar low-intensity smoking behaviours among Mexicans and Mexican-Americans led to similar all-cause mortality RRs, which were lower than those of smokers of other races/ethnicities in the United States.
